# Health Risk and Resilience Assessment with Respect to the Main Air Pollutants in Sichuan

**DOI:** 10.3390/ijerph16152796

**Published:** 2019-08-06

**Authors:** Junnan Xiong, Chongchong Ye, Tiancai Zhou, Weiming Cheng

**Affiliations:** 1 School of Civil Engineering and Architecture, Southwest Petroleum University, Chengdu 610500, China; 2 State Key Laboratory of Resources and Environmental Information System, Institute of Geographic Sciences and Natural Resources Research, CAS, Beijing 100101, China; 3 Synthesis Research Centre of Chinese Ecosystem Research Network, Key Laboratory of Ecosystem Network Observation and Modelling, Institute of Geographic Sciences and Natural Resources Research, Chinese Academy of Sciences, Beijing 100101, China; 4 College of Resources and Environment, University of Chinese Academy of Sciences, Beijing 100049, China

**Keywords:** air pollutants, health risk assessment, health resilience assessment, spatial inequality analysis

## Abstract

Rapid urbanization and industrialization in developing countries have caused an increase in air pollutant concentrations, and this has attracted public concern due to the resulting harmful effects to health. Here we present, through the spatial-temporal characteristics of six criteria air pollutants (PM_2.5_, PM_10_, SO_2_, NO_2_, CO, and O_3_) in Sichuan, a human health risk assessment framework conducted to evaluate the health risk of different age groups caused by ambient air pollutants. Public health resilience was evaluated with respect to the risk resulting from ambient air pollutants, and a spatial inequality analysis between the risk caused by ambient air pollutants and hospital density in Sichuan was performed based on the Lorenz curve and Gini coefficient. The results indicated that high concentrations of PM_2.5_ (47.7 μg m^−3^) and PM_10_ (75.9 μg m^−3^) were observed in the Sichuan Basin; these two air pollutants posed a high risk to infants. The high risk caused by PM_2.5_ was mainly distributed in Sichuan Basin (1.14) and that caused by PM_10_ was principally distributed in Zigong (1.01). Additionally, the infants in Aba and Ganzi had high health resilience to the risk caused by PM_2.5_ (3.89 and 4.79, respectively) and PM_10_ (3.28 and 2.77, respectively), which was explained by the low risk in these two regions. These regions and Sichuan had severe spatial inequality between the infant hazard quotient caused by PM_2.5_ (*G* = 0.518, *G* = 0.493, and *G* = 0.456, respectively) and hospital density. This spatial inequality was also caused by PM_10_ (*G* = 0.525, *G* = 0.526, and *G* = 0.466, respectively), which is mainly attributed to the imbalance between hospital distribution and risk caused by PM_2.5_ (PM_10_) in these two areas. Such research could provide a basis for the formulation of medical construction and future air pollution control measures in Sichuan.

## 1. Introduction

Air pollution is principally formed by a multifaceted mix of gaseous pollutants and suspended particulates [1], which affects atmospheric emission, absorption, and scattering of light. Recent research has reported that 3.7 million deaths due to exposure to outdoor air pollution in 2012. The number of deaths caused by ambient air pollutants is continuously rising, and will become the main environmental issue causing premature death by 2050 [2]. The continuous increase in human activities, such as urbanization and modernization, industrialization, human population, traffic density, and the number of motor vehicles, are the major contributors to the rising ambient air quality problems [3]. Air pollution has become a new threat to air quality [4], regional sustainable development, global climate change [5,6], and ecological suitability for human settlement [7]. In particular, air pollution continues to be a global environmental problem, and has been recognized as a severe public health risk [8]. Simultaneously, this health risk has drawn media coverage and captured public concern, motivating the government to take aggressive actions designed to control air pollution.

The sensitive parts of the lungs are susceptible to invasion by small, fine, and ultrafine ambient particles; these particles can exacerbate respiratory diseases such as bronchitis and emphysema, as well as existing heart disease, thus further leading to increased hospital admissions [9,10,11]. Additionally, respiratory and cardiovascular illnesses, especially perpetual obstructive aspiratory sicknesses, are some of the main causes of mortality in worldwide [12]. The above-mentioned issues and the enhanced emphasis on public health risk due to ambient air pollutants necessitate the estimation of the association between air pollution exposure and adverse public health effects. At present, public health risk assessments are performed by the human health risk assessment framework originating from the US Environmental Protection Agency (USEPA). This framework is a handy tool that is used to quantitatively calculate human health risk resulting from exposure to ambient air pollutants [13]. Further, inhalation of different atmospheric particulates by children and adults will lead to a different degrees of health risk, as investigated based on the above-mentioned framework [14]. In addition, the current application of this framework mainly considers people as having average exposure or continuous exposure [15].

Resilience is a term with various symbolic meanings and has become a popular theme across many disciplines, including health resilience, organizational resilience, psychological resilience, fund resilience, and urban resilience, etc. At present, urban resilience has been widely used, which has been applied to flash flood risk based on environmental and social indices (population density, building density, economic density, farmland density, road density, and urbanization rate, etc.) [16]. As to the risk caused by ambient air pollutants, one previous study defined the resilience to air pollution (mainly distributed in urban area) as the capacity of local stakeholders to formulate corresponding measures focused on hazard (or vulnerability) [2]. Furthermore, seven air quality resiliency factors for urban systems were defined by previous research, and included natural emissions mitigation, anthropogenic emissions, environmental policy, urban form and land-cover change, access to technology, geography, and socio-cultural dimensions of risk perception [17]. However, there are still limited studies that have used resilience to tackle air pollution, but health resilience can be seen with large and small risk, and the ability of people to recover from this risk [18].

Generally, although many studies have investigated the spatial and temporal patterns, chemical composition, potential sources, and effects of meteorological condition based on six criteria air pollutants, these studies have either ignored public health risk assessment [19,20,21,22,23,24,25], or only focused on a specific city or industrial region [3,15,26]. In the face of increasing risks caused by air pollutants, the public health resilience of different groups of people in Sichuan still needs a systematic and comprehensive analysis. Moreover, the reasonable spatial distribution of hospitals will play a vital role in people’s health, but this region lacks a study on spatial inequality analysis between the risk caused by air pollutants and hospital density. Therefore, the present study attempted to accomplish the following: (1) an assessment of the spatial-temporal patterns of six criteria air pollutants in Sichuan; (2) an evaluation of the risk caused by ambient air pollutants to different age groups on the spatial scale; (3) measurement of the public health resilience to the risk resulting from ambient air pollutants; and (4) analysis of the spatial inequality between the risk caused by air pollutants and hospital density in Sichuan.

## 2. Data and Methods

### 2.1. Study Area

**Figure 1:**
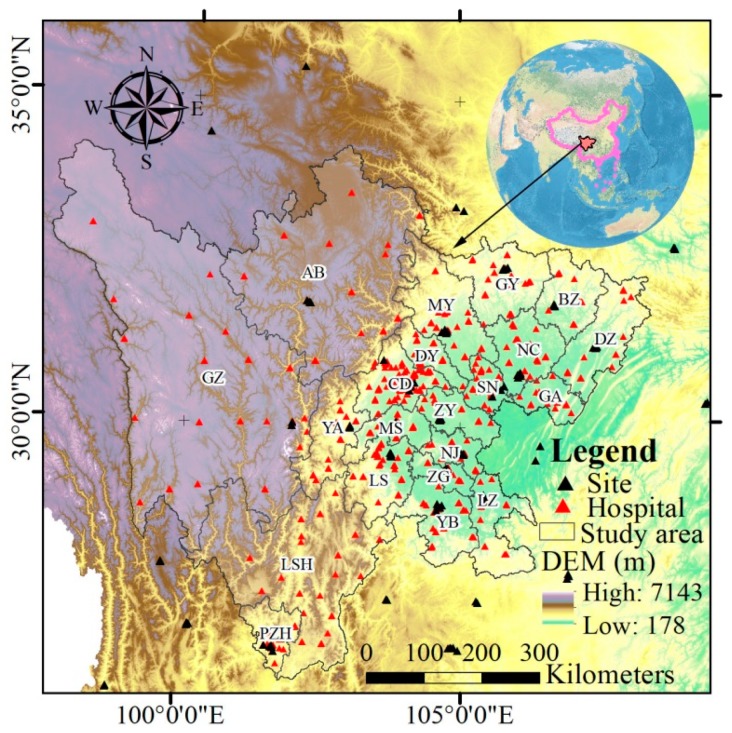
The study area.

Sichuan Province (26°03′–34°19′ N, 97°21′–108°33′ E), located in the transitional zone between the middle and lower reaches of Yangtze River and the Tibetan Plateau in southwest China, contains 21 cities (Figure 1 and Table 1). There are four types of topography: hills, mountains, plains, and plateaus. The annual average temperature is 16–18 °C and the precipitation is 1000–1200 mm in Sichuan Basin, although for northwestern Sichuan these values are 4–12 °C and 500–900 mm, respectively. In addition, Sichuan Province covers 486,000 km^2^ and has a population of 83.41 million; its gross domestic product (GDP) exceeded 4.06 trillion yuan at the end of 2018. Sichuan is thus an important province in southwest China. With the rapid development of the economy, the pace of urbanization has further increased, and the urbanization rate of the province’s permanent residents reached 50.4% in 2017 [27]. The specific conditions (e.g., geographical position, weather, and social and economic development) may exert a significant effect on air pollution characteristics.

### 2.2. Data

#### 2.2.1. Air Quality Data

In order to understand the characteristics of the six criteria air pollutants and the health risk assessment of main air pollutants in Sichuan, real-time hourly air pollutant data (PM_2.5_, PM_10_, SO_2_, NO_2_, CO, and O_3_) from January 2015 to December 2017 were obtained from the China National Environmental Monitoring Center [28], including 126 air-monitoring sites in and around Sichuan (Figure 1). The sites were set in each city according with the China Environmental Protection Standards HJ 655-2013 and HJ 193-2013 [29]. All of the air pollutants were monitored every 1 h except for O_3_, which was measured as an average over every 8 h. An advisable check was conducted at each site to remove faulty data before publishing these hourly air pollutant data [30].

For the spatial distribution of the six criteria air pollutants in different seasons, the seasonal concentrations of air pollutants were calculated by averaging the real-time hourly data in each site. On this basis, we used the inverse distance weight interpolation method in ArcGIS 10.2 software (Esri, Redlands, CA, USA) to get the air pollution raster data, and the resolution was 1 km × 1 km.

#### 2.2.2. Resilience Assessment Data

The spatial datasets were used to evaluate the public health resilience caused by air pollutants, including the hospital, economy, road density, land use, and night-time light data that related to various social coping capacity. Hospitals are an important indicator for the resilience assessment of public health, but the latitude and longitude data of hospital sites are not directly available at present. In this study, we collected hospital information from the Yao Zhi Data [31], which include information such as name, level, type, and address, and then we obtained the hospital’s longitude and latitude from the Baidu Map using the address information. This paper collected hospital data, but did not include sanitation stations, epidemic prevention and control centers, or community health care centers. In addition, we also selected economy, road density, land use, and night-time light except for hospital data, as these indicators can reflect the coping capacity to deal with the risks caused by air pollutants [32], and the original data sources are shown in Table 2. The data of pertussis vaccination rate was collected from Sichuan Health Statistics Yearbook (2017) [33].

In order to get the raster data of hospital density, the point density analysis was performed in ArcGIS 10.2 software. Previous studies have illuminated that the driving speeds during the trip to the hospital on a freeway, national highway, provincial highway, county highway, and village road are 100, 60, 50, 30, and 20 km/h, respectively, leading to a journey lasting about 1 h on these roads [34]. Therefore, we assumed that the service range of each hospital was 50 km, and took that as the radius for the point density analysis. The road density was calculated in ArcGIS 10.2 software based on the linear density analysis; the roads included freeways, national highways, provincial highways, county highways, and village roads. In addition, six land uses types were assigned values to each cell based on the intensity of human activity as 1, 2, 3, 4, 5, and 6 for unused land, water area, forest land, meadow, cultivated land, and urbanized area, respectively. For the night-time light, we calculated the three-year average as the indicator input. The final resolution of all the data was unified to 1 km.

### 2.3. Methods

To research the health risk and resilience assessment resulting from main air pollutants, and spatial inequality between the risk caused by ambient air pollutants and hospital density in Sichuan, we first analyzed the spatial characteristics and variation of the six criteria air pollutants. This section is organized as follows. Section 2.3.1 describes the health risk assessment of the main air pollutants. Section 2.3.2 presents the health resilience assessment. The Lorenz curve and Gini coefficient are presented in Section 2.3.3.

#### 2.3.1. Health Risk Assessment of Main Air Pollutants

Health risk assessment is a quantitative method for determining risks to human health caused by exposure to ambient air pollutants [14]. In the present study, it was revealed that PM_2.5_, PM_10_, SO_2_, NO_2_, CO, and O_3_ are harmful to public health and associated health risks [35]. Our study mainly explores the chronic (annual) exposure period, whereas the annual mean concentrations published by the ambient air quality standard (GB3095-2012) in China only include PM_2.5_, PM_10_, SO_2_, and NO_2_ (Table 3) [36]. Therefore, the health risk assessment takes the above-mentioned air pollutants (PM_2.5_, PM_10_, SO_2_, and NO_2_) as the research objects. In addition, the health risk assessment was performed among different age groups, namely, adults (19–75 years), children (6–12 years), and infants (birth to a year of age) [15]. 

Risk characterization is performed by the hazard quotient (HQ), which can reflect the probability of an adverse air pollutant occurring among healthy (or sensitive) individuals, thus avoiding the impacts of population density. An HQ ≤ 1.0 indicates a low risk, that is, the concentration of air pollutants is not likely to induce adverse health effects. An HQ > 1.0 indicates that the concentration of air pollutants may induce adverse effects on human health. The HQ was calculated as follows [14]:

(1) HQ=ADD/REL

where *REL* is the reference exposure level (REL). Based on the ambient air quality standard (GB3095-2012) [36], this paper takes the average value of Grade I and Grade II as the reference exposure level, as adopted by the previous research [37]. *ADD* is the daily dose, and is calculated as follows [38]:

(2) ADD=(CA×IR×ED)/(BW×AT)

where *CA* denotes the concentration of air pollutants (μg m^−3^), *IR* denotes the average values of inhalation rate (m^3^ day^−1^), *ED* denotes the exposure duration (day), *BW* denotes the average weight (kg), and *AT* denotes the averaging time (day). All parameter standards are derived from the US Environmental Protection Agency [39], which are shown in Table 4.

#### 2.3.2. Health Resilience Assessment

In this paper, the health resilience assessment is defined as the capacity of public health to recover from the risks caused by ambient air pollutants [18]. Based on the result of health risk assessment, we focused health resilience assessment on air pollutants that pose a high risk to public health, and the process is as follows: (1) calculation of the social coping capacity; and (2) calculation of health resilience to the risk caused by air pollutants.

1. Calculation of social coping capacity

In order to quantify social coping capacity, principal component analysis was selected to assist us because this method is the oldest multivariate technique and can reflect important information from the original indicators [40,41]. In this study, the standardized results of the hospital, GDP, road density, land use, and night-time light were transformed into two principal components in ArcGIS 10.2 software. The number of principal components was determined based on previous studies [42], and the contribution rates of these two principal components to the original indicators information were 0.742 and 0.224, respectively. The social coping capacity is calculated as follows [43]:

(3) $$S=\sum_{i=1}^{n}C_{i}P_{i}$$

where *n* is the number of principal components, *C_i_* is the contribution rate of principal component *i*, and *P_i_* is the standardized result of principal component *i*.

2. Calculation of health resilience

Health resilience is largely determined by the social coping capacity and the risks caused by air pollutants: it is the existence of high social coping capacity and low risks that can lead to high health resilience. The health resilience to the risk caused by air pollutants can be expressed as follows [44]:

(4) $$R=S_{s}/HQ_{s}$$

where *R* is the health resilience to the risk caused by air pollutants, *S_s_* is the standardized result of social coping capacity, and *HQ_s_* is the standardized result of HQ. An *R* that is ≤1.0 indicates a low health resilience, that is, the social coping capacity is lower than risks caused by air pollutants. Meanwhile, *R* > 1.0 indicates a high health resilience, that is, the social coping capacity can deal with risks caused by air pollutants.

#### 2.3.3. Lorenz Curve and Gini Coefficient

**Figure 2:**
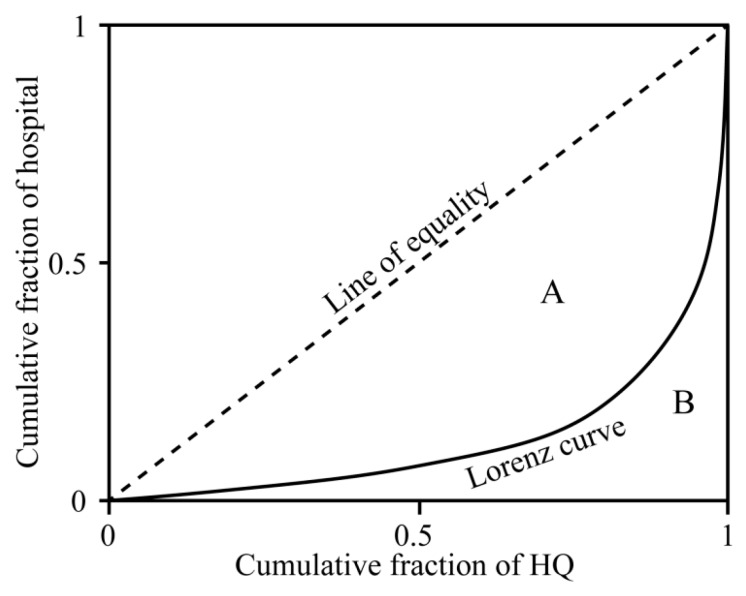
Lorenz curve diagram.

The Lorenz curve is a model used in economics to measure the equitability of the distribution of social resources across the population, and the Gini coefficient can quantify the extent of inequality between two variables [45]. Previous studies identified the regional disparity of emission distribution of air pollutants and socio-economic condition by the Lorenz curve and Gini coefficient [46]. In this study, to calculate the spatial inequality between the risk caused by air pollutants and hospital density (risk is represented by HQ, hospital density is quantified by density analysis result of hospital point), this relationship is performed as a curve with a concave slope (Figure 2). The perfect equality line is the upper limit of the Lorenz curve, and a greater gap between the perfect equality line and Lorenz curve that leads to greater distribution inequality. The Gini coefficient evaluates the extent of inequality by comparing the area between the Lorenz curve and the perfect equality line (*A*) and the area under the equality line (*A* + *B*) (Figure 2) [47]:

(5) $$G=\frac{A}{A+B}$$

where *G* ranges from 0 to 1; *G* = 0 indicates that the risk and hospital density are in balance. The larger Gini coefficient leads to greater spatial imbalance between the risk and hospital density. The Lorenz curve and Gini coefficient were obtained using the package “lawstat” in R software (R Core Development Team, R Foundation for Statistical Computing, Vienna, Austria).

## 3. Results

### 3.1. Spatial and Temporal Patterns of Six Criteria Air Pollutants

**Figure 3:**
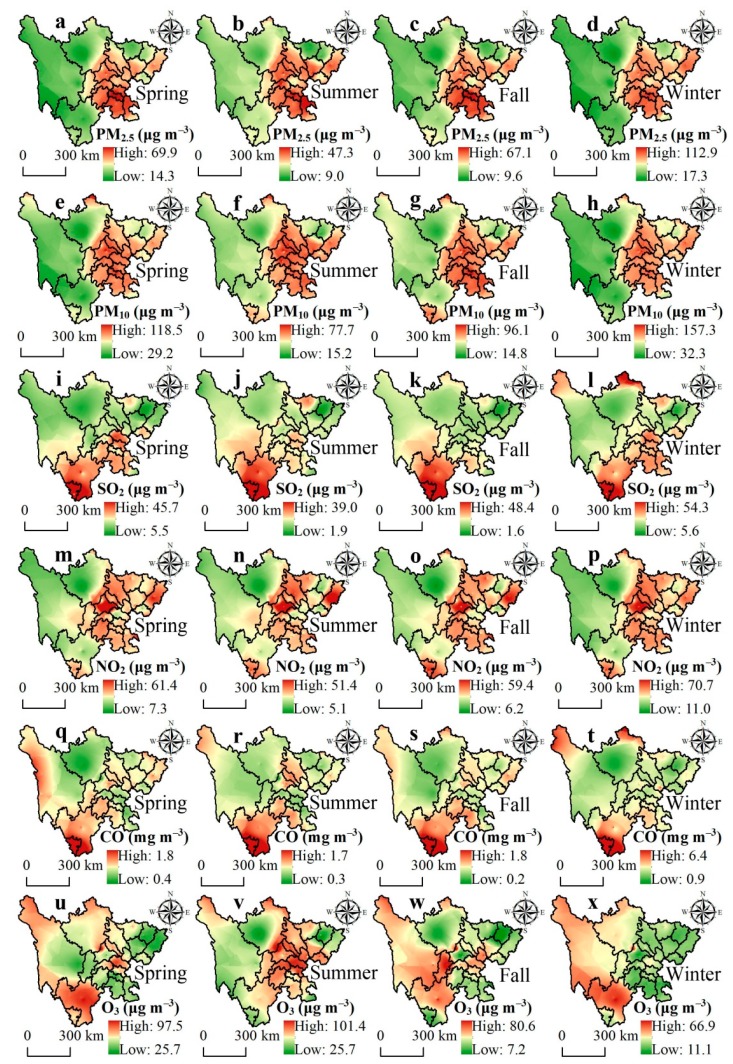
Spatial patterns of the six criteria air pollutants showing the seasonal mean concentrations from 2015 to 2017: (**a**) PM_2.5_ *, (**e**) PM_10_ *, (**i**) SO_2_ *, (**m**) NO_2_ *, (**q**) CO ^#^, and (**u**) O_3_ * in spring; (**b**) PM_2.5_ *, (**f**) PM_10_ *, (**j**) SO_2_ *, (**n**) NO_2_ *, (**r**) CO ^#^, and (**v**) O_3_ * in summer; (**c**) PM_2.5_ *, (**g**) PM_10_ *, (**k**) SO_2_ *, (**o**) NO_2_ *, (**s**) CO ^#^, and (**w**) O_3_ * in fall; (**d**) PM_2.5_ *, (**h**) PM_10_ *, (**l**) SO_2_ *, (**p**) NO_2_ *, (**t**) CO ^#^, and (**x**) O_3_ * in winter. Units: * μg m^−3^; ^#^ mg m^−3^.

According to the spatial distribution of six criteria air pollutants in Sichuan from spring to winter (Figure 3), we discovered that the PM_2.5_ and PM_10_ have the same pattern, with the maximum distribution in Sichuan Basin, and the minimum distribution in the northwest part of Sichuan (Figure 3a–h). Furthermore, the highest concentrations of PM_2.5_ and PM_10_ occurred in winter, and ranged from 17.3 to 112.9 μg m^−3^ and from 32.3 to 157.3 μg m^−3^, respectively. According to Figure 3i–l,q–t, Panzhihua had the highest concentrations of SO_2_ and CO in spring (29.9 μg m^−3^ and 1.3 mg m^−3^, respectively), summer (28.2 μg m^−3^ and 1.3 mg m^−3^, respectively), fall (36.6 μg m^−3^ and 1.6 mg m^−3^, respectively), and winter (40.6 μg m^−3^ and 4.7 mg m^−3^, respectively). The concentration ranges of NO_2_ were 7.3–61.4 μg m^−3^, 5.1–51.4 μg m^−3^, 6.2–59.4 μg m^−3^, and 11.0–70.4 μg m^−3^, in spring, summer, fall, and winter, respectively (Figure 3m–p); the maximum values were primarily distributed in Chengdu. However, the highest O_3_ concentration occurred in summer (25.7–101.4 μg m^−3^), and the lowest concentration occurred in winter (11.1–66.9 μg m^−3^) (Figure 3v,x). We also found that in summer the maximum O_3_ concentration was chiefly located in Sichuan Basin, while the minimum concentration was in Bazhong.

### 3.2. Health Risk Assessment of Chronic Exposure Across the Population

**Figure 4:**
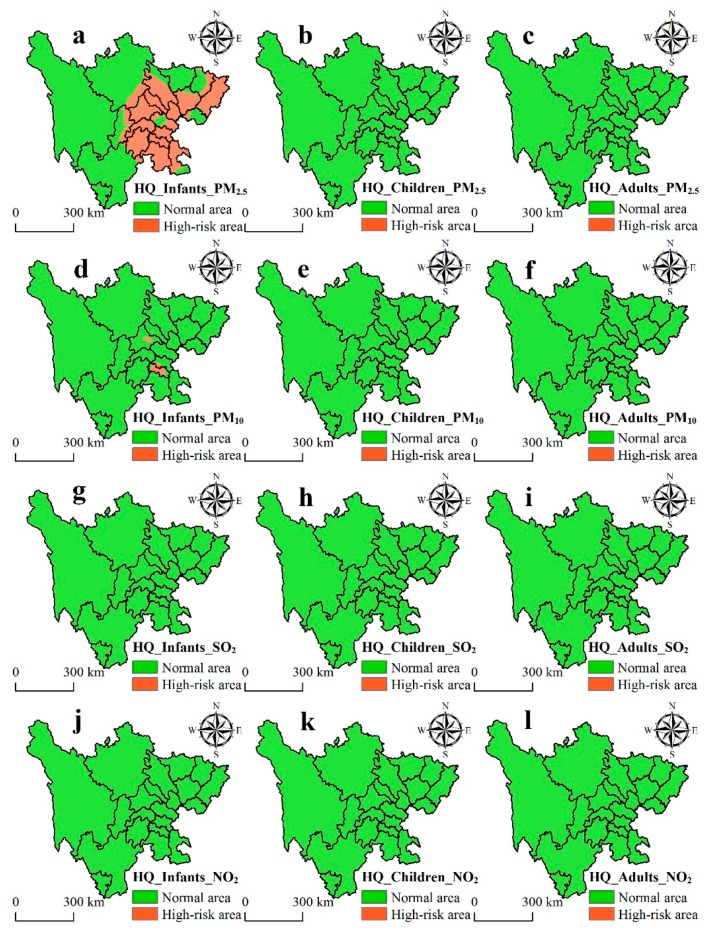
Risk assessment of chronic exposure to main air pollutants in Sichuan. (**a**–**c**)/(**d**–**f**)/(**g**–**i**)/(**j**–**l**) represent hazard quotient for infants, children, and adults for PM_2.5_ (HQ_Infants_PM_2.5_, HQ_Children_PM_2.5_, and HQ_Adults_PM_2.5_)/PM_10_ (HQ_Infants_PM_10_, HQ_Children_PM_10_, and HQ_Adults_PM_10_)/SO_2_ (HQ_Infants_SO_2_, HQ_Children_SO_2_, and HQ_Adults_SO_2_)/NO_2_ (HQ_Infants_NO_2_, HQ_Children_NO_2_, and HQ_Adults_NO_2_), respectively.

To better understand the health risk assessment of chronic (annual) exposure in Sichuan, the risk caused by PM_2.5_, PM_10_, SO_2_, and NO_2_ are shown in Figure 4. For infants, according to Figure 4a, the high-risk values of PM_2.5_ are mainly distributed in Leshan (1.25), Meishan (1.18), Mianyang (1.05), Nanchong (1.09), Neijiang (1.35), Chengdu (1.23), Dazhou (1.15), Deyang (1.19), Suining (1.08), Yibin (1.34), Ziyang (1.06), Zigong (1.62), and Luzhou (1.21). The high-risk values of PM_10_ occurred mainly in Zigong (1.01) (Figure 4d). However, no high-risk values of SO_2_ and NO_2_ were observed in Sichuan (Figure 4g,j). For children and adults, there were no high-risk areas for PM_2.5_, PM_10_, SO_2_, and NO_2_ (Figure 4b,c,e,f,h,i,k,l).

### 3.3. Health Resilience Assessment of Infants for the Risk Caused by PM_2.5_ and PM_10_

**Figure 5:**
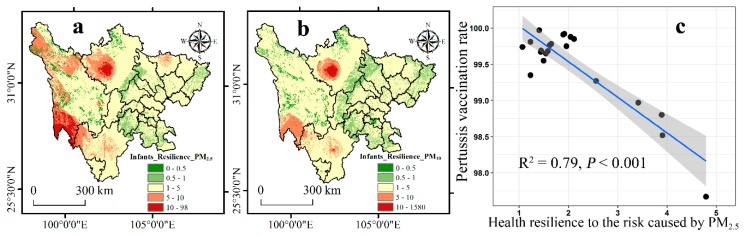
The health resilience of infants to the risk caused by (**a**) PM_2.5_ (Infants_Resilience_PM_2.5_) and (**b**) PM_10_ (Infants_Resilience_PM_10_); (**c**) the correlation between the pertussis vaccination rate and average health resilience of infants to the risk caused by PM_2.5_ in 21 cities in Sichuan.

On the basis of the risk caused by PM_2.5_ and PM_10_ to the infants, the health resilience assessment is shown in Figure 5. For the risk caused by PM_2.5_, the high values of health resilience were mainly distributed in the southwest of Aba and the south of Ganzi, and the low values were around the borders of Aba and Chengdu, Deyang, and Mianyang, as well as in Leshan, Yibin, Zigong, Dazhou and Luzhou (Figure 5a). As to the average health resilience of infants to the risk caused by PM_2.5_, the cities of Ganzi (4.79), Liangshan (3.91), Aba (3.89), and Guangyuan (3.42) have high health resilience. The health resilience of infants to the risk caused by PM_10_ had the same pattern as PM_2.5_, but with Yaan having a large area of low health resilience (Figure 5b).

Since research assessing the health resilience to the risk caused by ambient air pollutants is in progress, there is a current lack of studies that verify the validity of health resilience assessment. Hence, a correlation analysis was conducted on the pertussis vaccination rate and average health resilience of infants to the risk caused by PM_2.5_ in 21 cities in Sichuan. There is a correlation coefficient of 0.79, which indicates that a better health resilience to the risk caused by air pollutants will lead to weaker vaccination awareness (Figure 5c).

### 3.4. Spatial Inequality Analysis between Risk Caused by Air Pollutants and Hospital Density

**Figure 6:**
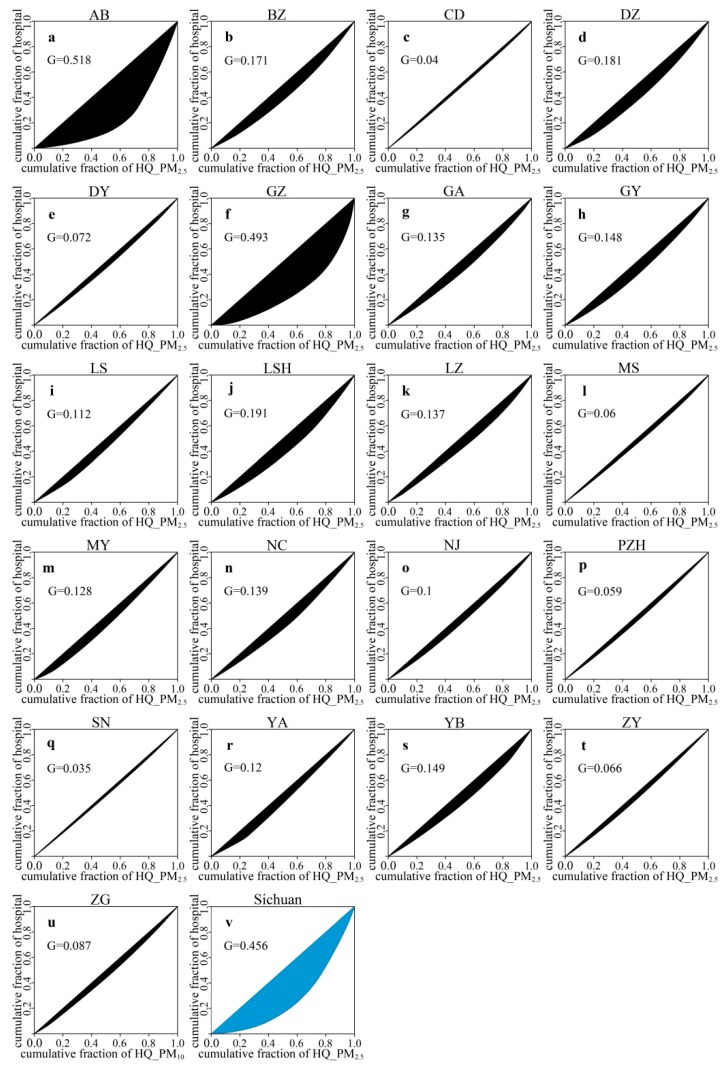
Spatial inequality analysis between the infant hazard quotient caused by PM_2.5_ (HQ_PM_2.5_) and hospital density: (**a**) Aba, (**b**) Bazhong, (**c**) Chengdu, (**d**) Dazhou, (**e**) Deyang, (**f**) Ganzi, (**g**) Guangan, (**h**) Guangyuan, (**i**) Leshan, (**j**) Liangshan, (**k**) Luzhou, (**l**) Meishan, (**m**) Mianyang, (**n**) Nanchong, (**o**) Neijiang, (**p**) Panzhihua, (**q**) Suining, (**r**) Yaan, (**s**) Yibin, (**t**) Ziyang, (**u**) Zigong, and (**v**) Sichuan.

**Figure 7:**
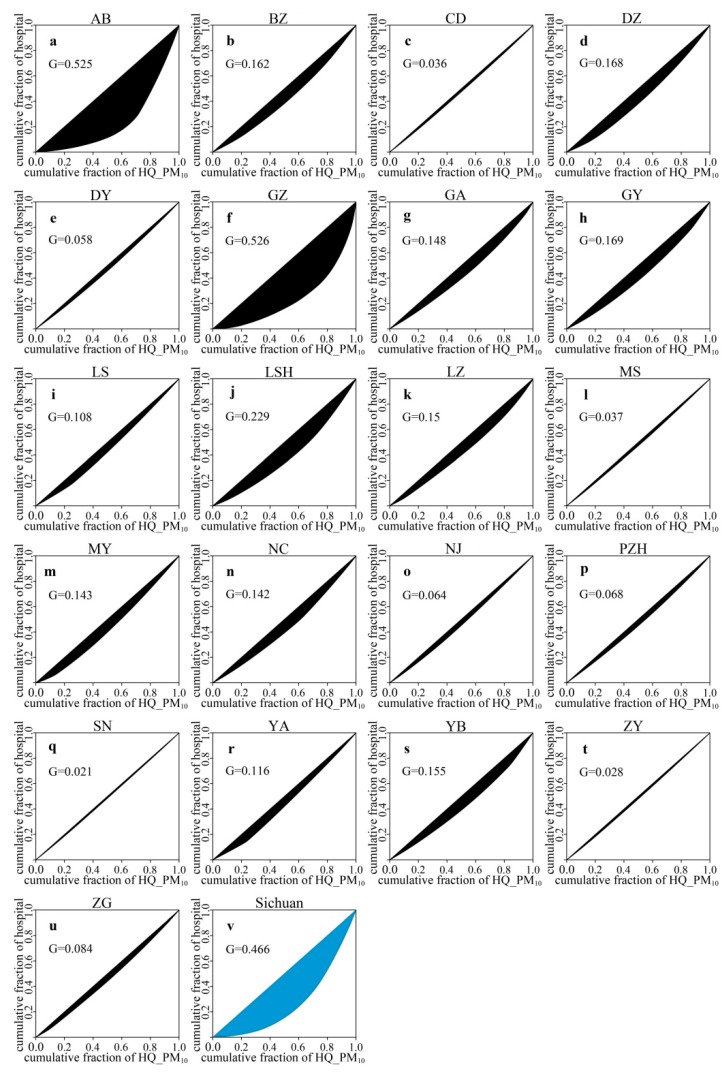
Spatial inequality analysis between the infant hazard quotient caused by PM_10_ (HQ_PM_10_) and hospital density: (**a**) Aba, (**b**) Bazhong, (**c**) Chengdu, (**d**) Dazhou, (**e**) Deyang, (**f**) Ganzi, (**g**) Guangan, (**h**) Guangyuan, (**i**) Leshan, (**j**) Liangshan, (**k**) Luzhou, (**l**) Meishan, (**m**) Mianyang, (**n**) Nanchong, (**o**) Neijiang, (**p**) Panzhihua, (**q**) Suining, (**r**) Yaan, (**s**) Yibin, (**t**) Ziyang, (**u**) Zigong, and (**v**) Sichuan.

As shown in Figure 6 and Figure 7, we conducted a spatial inequality analysis between risk caused by PM_2.5_ (PM_10_) and hospital density. High spatial inequalities level between risk caused by PM_2.5_ and hospital density mainly occurred in Aba (*G* = 0.518) and Ganzi (*G* = 0.493), whereas low spatial inequalities were chiefly concentrated in Meishan (*G* = 0.06), Chengdu (*G* = 0.04), Deyang (*G* = 0.072), Panzhihua (*G* = 0.059), Suining (*G* = 0.035), Ziyang (*G* = 0.066), and Zigong (*G* = 0.087) (Figure 6a,c,e,f,l,p,q,t,u). In addition, we found that the entire province of Sichuan also remained high spatial inequality (*G* = 0.456) (Figure 6v). The spatial inequality between risk caused by PM_10_ and hospital density had the same trend as PM_2.5_: Aba (*G* = 0.525) and Ganzi (*G* = 0.526) had high spatial inequalities, while low spatial inequalities primarily occurred in Meishan (*G* = 0.037), Neijiang (*G* = 0.064), Chengdu (*G* = 0.036), Deyang (*G* = 0.058), Panzhihua (*G* = 0.068), Suining (*G* = 0.021), Ziyang (*G* = 0.028), and Zigong (*G* = 0.084) (Figure 7a,c,e,f,l,o–q,t,u). Simultaneously, Sichuan also had high spatial inequality between risk caused by PM_10_ and hospital density had the same trend as PM_2.5_ (*G* = 0.466) (Figure 7v).

## 4. Discussion

### 4.1. Spatial and Temporal Patterns of Air Pollutants

Our study identified that the Sichuan Basin had high concentrations of PM_2.5_ and PM_10_, and low concentrations were mainly distributed in the northwestern Sichuan region (Figure 3a–h). A few studies have revealed that the Sichuan Basin had high populations, dense urban traffic, rapid urban expansion, and large exhaust emissions, which are the direct causes of the high concentration of suspended particles in this region [29,48]. Furthermore, a previous study reported that industrial emissions contributed 27% to PM_2.5_ concentration in Wuhan during 2011–2012 [49]. However, the northwestern Sichuan region is a high-altitude area, and the local residents mainly rely on grazing [50], and thus low industrial activity might be the main reason for the PM_2.5_ and PM_10_ patterns. We also found that Panzhihua has the highest concentrations of SO_2_ and CO (Figure 3i–l,q–t). Usually, secondary industry plays an important role in the high concentration of SO_2_ and CO. For example, a previous study showed that steel manufacturing contributed 24% and 30% to SO_2_ and CO concentrations, respectively, in Tianjin [51]. Therefore, the high concentrations of SO_2_ and CO in Panzhihua are attributed to the more developed steel industry in this region [52]. As to NO_2_, Chengdu had the highest concentration (Figure 3m–p), this region had 3128 thousand vehicles and a GDP of 1005.7 billion yuan in 2015 [29], which we consider to have contributed to the high emissions of NO_2_ that was supported by the previous research [53].

The spatial characteristics of PM_2.5_, PM_10_, SO_2_, NO_2_, and CO were basically the same in each season, and the highest concentrations occurred in winter (Figure 3a–t). This pattern is a typical characteristic in East Asia [54], the concentration of air pollutants in winter are chiefly affected by emission sources and meteorological conditions. Usually, Sichuan has frequent stagnant weather conditions in winter, characterized by slow winds, little precipitation, and shallow mixing layers; this special condition can capture the air pollutants emitted by local sources (or air pollutants transported from other regions) and elevates winter pollutant concentrations [55]. Coal combustion is another important factor influencing the air pollutant patterns in winter. Previous studies have reported that emissions of CO (32%), NO_2_ (70%), SO_2_ (90%), and PM_2.5_ (97%) were attributed to coal combustion [56]. Regarding O_3_, which showed different seasonal distributions, the lowest and highest concentrations occurred in winter and summer, respectively. In summer, the high concentration of O_3_ was mainly distributed in Sichuan Basin (Figure 3u–x); research based on fossil fuels and shortwave radiation provided direct evidence that the high fossil fuel consumption and strong solar radiation in the summer significantly influenced the O_3_ concentration in this region [57,58]. In winter, the highest concentration of O_3_ was concentrated in Liangshan; a previous study confirmed that the increase in O_3_ concentration in this area is mainly caused by the secondary conversion of inorganic ions [59].

### 4.2. Health Risk Assessment of the Population

Air pollutants remain a global environmental threat and cause a public health risk. Some researchers assumed that health risk from exposure to ambient air pollutants can happen below or at the concentrations allowed by the international and national air quality standards. Findings in this study revealed that, for infants, Leshan (*HQ* = 1.25), Meishan (*HQ* = 1.18), Mianyang (*HQ* = 1.05), Nanchong (*HQ* = 1.09), Neijiang (*HQ* = 1.35), Chengdu (*HQ* = 1.23), Dazhou (*HQ* = 1.15), Deyang (*HQ* = 1.19), Suining (*HQ* = 1.08), Yibin (*HQ* = 1.34), Ziyang (*HQ* = 1.06), Zigong (*HQ* = 1.62), and Luzhou (*HQ* = 1.21) had high health risks caused by PM_2.5_ (Figure 4a). Evidence from the PM_2.5_ concentration shows the ambient concentration (47.7 μg m^−3^) in the Sichuan Basin exceeded the Grade II standard (35 μg m^−3^). Furthermore, previous research revealed that respiratory infection disease in infants (in the absence of congenital diseases) are positively correlated with PM_2.5_ concentration, and they also reported that the Hengshui People’s Hospital received a total of 160 infants with diseases related to air pollutants from June 2014 to January 2015 [60]. This study further revealed that the high health risk of infants caused by PM_10_ occurred mainly in Zigong (*HQ* = 1.01) (Figure 4d). Zigong had the highest average annual concentration of PM_10_ (87.0 μg m^−3^) in Sichuan and was higher than the Grade II standard (70 μg m^−3^). Moreover, it was confirmed that the concentration of air pollutants on the ground tends to be higher [61]; hence, infants are more likely to inhale PM, which leads to higher health risks. A previous study also reported that a 10 μg m^−3^ increase in PM_10_ concentration can lead to an increase in hospital admissions for ischemic heart disease and congestive heart failure [62]. Our study showed that SO_2_ and NO_2_ did not pose high health risks for infants (Figure 4g,j); however, another researcher revealed that Chengdu had high health risks caused by NO_2_ in 2014 and 2015 [38]. The low risk after 2015 might be supported by Chengdu’s efforts to improve air quality in recent years [63]. Regarding children and adults, the PM_2.5_, PM_10_, SO_2_, and NO_2_ did not cause high health risk in Sichuan (Figure 4b,c,e,f,h,i,k,l). It has been confirmed that infants have a higher susceptibility to ambient air pollutants than children and adults. Furthermore, infants are considered a health risk group for numerous reasons, chiefly due to the relatively higher amount of air inhalation (as their air intake per weight unit in a resting state is twice that of an adult), and the fact that the lungs and immune system are not yet fully developed [13].

### 4.3. The Health Resilience Assessment and Spatial Inequality Analysis

The health resilience assessment clearly presented the health resilience of infants to the risk caused by PM_2.5_ and PM_10_, and our spatial inequality analysis revealed the spatial inequality between risk caused by PM_2.5_ (PM_10_) and hospital density in Sichuan. Our study demonstrated that a high health resilience to the risk caused by PM_2.5_ was mainly distributed in Aba and Ganzi (Figure 5a). These cities have approximately 53.7% and 61.7% natural grassland, respectively [64,65]; furthermore, local residents mainly rely on grazing, and there are lower levels of industrial emissions and automobile exhaust in these two regions [50]. In this condition, air pollution did not pose a high risk, and therefore the people living there have a high level of health resilience to the low risk caused by PM_2.5_. Additionally, the *Ten-Year Action Plan for Health Development in Ethnic Regions of Sichuan Province*, a significant plan for the medical development of ethnic areas launched in 2011, improved the medical conditions and promoted health resilience in Aba and Ganzi [66]. However, there is still a high spatial inequality between risk caused by PM_2.5_ and hospital density in Aba (*G* = 0.518) and Ganzi (*G* = 0.493) (Figure 6a,f), which indicates that these two regions need to continue to strengthen their medical systems with a reasonable spatial distribution. In addition, we further revealed that low health resilience to the risk caused by PM_2.5_ was principally concentrated in Leshan, Yibin, Zigong, Dazhou, and Luzhou (Figure 5a). A previous study reported that Sichuan Basin had a high level of PM_2.5_ concentration, and a cumulative concentration of PM_2.5_ with a three-day lag increased by 1 inter-quartile range (IQR) will led to the number of asthma patients increasing by 3.27% [67]. Additionally, Leshan (47.31%), Yibin (45.10%), Zigong (47.88%), Dazhou (40.87%), and Luzhou (46.08%) have a lower urbanization rates than Chengdu (71.47%) [68], and their spatial inequalities between risk caused by PM_2.5_ and hospital density were greater than that of Chengdu (Figure 6c,d,i,k,s,u), which together determined that the low health resilience to the risk caused by PM_2.5_ in the above-mentioned cities. Furthermore, we also found that the health resilience of infants to the risk caused by PM_10_ was the same as that for PM_2.5_, which is supported by previous researches based on the correlation analyses between PM_10_ and PM_2.5_ [35,69].

### 4.4. Uncertainties and Limitations

Our results showed that PM_2.5_ and PM_10_ pose a high risk to infants. The high risk caused by PM_2.5_ is mainly distributed in Sichuan Basin, and that caused by PM_10_ principally occurs in Zigong. Additionally, the high health resilience of infant to the risks caused by PM_2.5_ and PM_10_ were chiefly concentrated in Aba and Ganzi. Although uncertainties exist in human health risk and resilience assessments, their application have found that the usefulness in providing a consistent and quantitative method to systematically evaluate public health risk and resilience caused by air pollutants, and inform decisions for air pollution control. Human health resilience assessment used in this study is conservative as it includes many social factors that are built into the social coping capacity. To address these uncertainties, we verified the validity of the health resilience assessment by analyzing the correlation between the pertussis vaccination rate and average health resilience of infants to the risk caused by PM_2.5_ in 21 cities in Sichuan. 

The findings in this study should be considered in light of the following limitations. The public health risk and resilience assessment in this study adopted different age groups of people as the unit of research rather than population density. The AT used in this study for adult group is the average value (30 × 365), which may bring errors to the risk assessment results of young people and the elderly. As to the validity of the health resilience assessment, we measured the correlation between the pertussis vaccination rate and average health resilience of infants to the risk caused by PM_2.5_ in 21 cities in Sichuan, but the local economic and quality of public health service play an important role in controlling the pertussis vaccination rate. We assumed that the service capabilities of all hospitals are the same. Also, public health resilience refers to the ability of the public health to recover from the risk caused by air pollutants. The high social coping capacity and low risks caused by air pollutants can determine the high public health resilience. 

The strengths of our study are worth mentioning. We described the public health risk and resilience associated with human exposure to PM_2.5_, PM_10_, SO_2_, and NO_2_ on the spatial scale in Sichuan, which can inform the decisions for air pollutant control. In addition, the spatial inequality analysis between risk caused by air pollutants and hospital density can reflect the spatial distribution of medical care in a region, thus providing a certain reference for the government to build medical services.

## 5. Conclusions

Ambient air pollution is harmful to public health. Our study focused on the spatial-temporal characteristics of air pollutants and the public health risk and resilience assessment with respect to the main air pollutants. We also analyzed the spatial inequality between risk caused by air pollutants and hospital density in Sichuan. High concentrations of PM_2.5_ and PM_10_ were mainly concentrated in Sichuan Basin, which posed a high risk to infants. The high risk caused by PM_2.5_ was mainly distributed in Sichuan Basin, and that caused by PM_10_ principally occurred in Zigong. Additionally, Aba and Ganzi had high health resilience of infants to the risks caused by PM_2.5_ and PM_10_, while low health resilience was chiefly concentrated in Leshan, Yibin, Zigong, Dazhou, and Luzhou. Furthermore, Sichuan, Aba, and Ganzi had serious spatial inequality between risk caused by PM_2.5_ (PM_10_) and hospital density.

This study attempted to evaluate the health resilience to the main air pollutants in Sichuan, but it is conservative as it includes limited social factors (hospital, GDP, road density, land use, and night-time light). However, the identification of the potential of air pollution to pose public health hazards and the measured public health resilience to the risks caused by air pollutants provide a valuable contribution for environmental departments, and will aid in the creation of more concrete policies to protect and prolong human lives. On the basis of the results of this study, policymakers should strengthen existing legislation to limit the release of PM_2.5_ and PM_10_ in Sichuan Basin, especially in Zigong; moreover, Aba and Ganzi need to continue to strengthen their medical systems, with a reasonable spatial distribution.

## Figures and Tables

**Table 1:** The 21 cities in Sichuan Province.

City	Abbreviation	City	Abbreviation	City	Abbreviation
Aba	AB	Guangyuan	GY	Neijiang	NJ
Bazhong	BZ	Leshan	LS	Panzhihua	PZH
Chengdu	CD	Liangshan	LSH	Suining	SN
Dazhou	DZ	Luzhou	LZ	Yaan	YA
Deyang	DY	Meishan	MS	Yibin	YB
Ganzi	GZ	Mianyang	MY	Ziyang	ZY
Guangan	GA	Nanchong	NC	Zigong	ZG

**Table 2:** Source and resolution of resilience assessment data.

Indicators	Data Source	Resolution	Data Type	Time
Hospital	China: Yao Zhi Data	–	–	2015
GDP	China: Resource and Environment Data Cloud Platform	1 km × 1 km	Raster data	2015
Roads	China: National Earth System Science Data Sharing Infrastructure	1:250,000	Vector data	2016
Land use	China: Resource and Environment Data Cloud Platform	1 km × 1 km	Raster data	2015
NPP-VIIRS Night-time light	USA: National Oceanic and Atmospheric Administration	500 × 500 m	Raster data	2015–2017

**Table 3:** Ambient air quality standard in China.

Air Pollutants	Annual Mean Concentration		Unit
	Grade I	Grade II	
PM_2.5_	15	35	μg m^−3^
PM_10_	40	70	μg m^−3^
SO_2_	20	60	μg m^−3^
NO_2_	40	40	μg m^−3^

**Table 4:** Parameters for health risk assessment of main air pollutants through the inhalation pathway. *IR* is the average values of inhalation rate, *ED* is the exposure duration, *BW* is the average weight, and *AT* is the averaging time.

Exposed Group	*IR* (m^3^ day^−1^)	*ED* (day)	*BW* (kg)	*AT* (day)
Infant	6.8	1 × 350	11.3	1 × 365
Child	13.5	12 × 350	45.3	12 × 365
Adult	13.3	30 × 350	71.8	30 × 365
